# 912. Physical Morbidity After Burns: Understanding Long-term Sequelae – a Burn Model Systems Study

**DOI:** 10.1093/jbcr/irag033.437

**Published:** 2026-04-06

**Authors:** Eloise W Stanton, Kara McMullen, Jeffrey C Schneider, Haig A Yenikomshian

**Affiliations:** Division of Plastic and Reconstructive Surgery, Keck School of Medicine, University of Southern California, Los Angeles, CA; Department of Rehabilitation Medicine, University of Washington, Seattle, WA; Spaulding Rehabilitation Hospital, Mass General Brigham, Harvard Medical School, Boston, MA; Division of Plastic and Reconstructive Surgery, Keck School of Medicine, University of Southern California, Los Angeles, CA

## Abstract

**Introduction:**

Burn injury survivors often face persistent physical and psychological complications long after acute recovery. Although functional and psychosocial outcomes have been increasingly studied, the long-term physical morbidities of burn injury remain underrecognized. Importantly, payors and health systems rarely view burns as a chronic condition, despite mounting evidence that survivors experience enduring sequelae requiring ongoing care. This gap in recognition limits resource allocation, follow-up care, and support for patients navigating long-term recovery. To address this, the present study aimed to characterize morbidity trends among adult burn survivors and to evaluate how chronic complications influence patient-reported outcomes two years after injury.

**Methods:**

Data were drawn from a large multi-center burn database. Adults (≥18 years) with burns sustained after 2015 who had review-of-systems (ROS) data and PROMIS outcomes at 12 months were included. Descriptive statistics summarized demographics, injury characteristics, and morbidity prevalence. Linear regression models assessed associations between chronic conditions (composite ROS variable) and PROMIS outcomes at 12 months, adjusting for age, sex, race/ethnicity, insurance, %TBSA, inhalation injury, and site.

**Results:**

Of 1765 eligible participants, 705 met inclusion criteria. The cohort was predominantly male (68%) and White (79%), with mean age 47.5 years and median TBSA 10%. At 12 months, chronic sequelae were common: neuropathic symptoms (65%), joint pain (41%), memory difficulty (27%), cold intolerance (32%), and vision problems (16%). Chronic conditions were associated with poorer PROMIS outcomes, including lower physical function (β = –5.37, *p*<.001) and social participation (β = –6.18, *p*<.001), and greater anxiety, depression, fatigue, sleep disturbance, and pain interference (all *p*<.001). Higher TBSA predicted worse physical and social outcomes, while female sex and public insurance were linked with poorer psychosocial health.

**Conclusions:**

Burn survivors experience a high burden of chronic morbidity that affects quality of life at two years. These complications predict impaired function and psychosocial distress, independent of burn size and demographics. Findings highlight the need for long-term, multidisciplinary follow-up and targeted interventions to improve survivorship.

**Applicability of Research to Practice:**

Burn survivors face a high burden of chronic physical and psychosocial complications that significantly impair long-term quality of life. Incorporating structured screening and multidisciplinary follow-up into routine care can help clinicians identify persistent morbidities early and provide targeted interventions to improve survivorship outcomes.

**Funding for the study:**

The contents of this project were developed under a grant from the National Institute on Disability, Independent Living, and Rehabilitation Research (NIDILRR grant numbers 90DPBU0007, 90DPBU0008, 90DPBU0009). NIDILRR is a Center within the Administration for Community Living (ACL), Department of health and Human Services (HHS). The contents of this manuscript do not necessarily represent the policy of NIDILRR, ACL, or HHS, and you should not assume endorsement by the Federal Government.

